# Case report of atypical undernutrition of hypoproteinemia type

**DOI:** 10.1515/biol-2022-0766

**Published:** 2023-11-29

**Authors:** Qun-Ru Wang, Jun Long, Chen-Cheng Wang, Ji-Lei Hu, Ning Lin, Shan-Hong Tang

**Affiliations:** Department of Gastroenterology, General Hospital of Western Theater Command of Chinese PLA, Chengdu 610083, China; Department of Nutrition, General Hospital of Western Theater Command of Chinese PLA, Chengdu 610083, China

**Keywords:** cavernous transformation of the portal vein, hypoproteinemia, portal thrombosis, undernutrition

## Abstract

Albumin and prealbumin serve as vital markers reflecting hepatic synthesis activity and overall body nutrient status. Hypoproteinemia can result from various etiological factors, with reduced blood inflow into the liver due to portal vein thrombosis being one such cause. However, literature addressing this specific association remains limited. This report presents an atypical case of malnutrition involving a patient who experienced prolonged hypoproteinemia attributable to a gradual decline in hepatic blood perfusion caused by progressive portal thrombosis and cavernous transformation of the portal vein (CTPV). The case encompasses an in-depth analysis of the factors contributing to undernutrition, the etiology and diagnosis of hypoproteinemia, and its clinical implications. Vigilance for the presence of hypoproteinemia is essential in the management of patients afflicted by progressive portal vein thrombosis complicated by CTPV. Timely and effective interventions aimed at rectifying hypoproteinemia can significantly enhance clinical outcomes. Moreover, reduced hepatic blood flow should be considered a plausible underlying cause in cases of unexplained hypoproteinemia, warranting thorough evaluation. This case underscores the importance of recognizing the intricate interplay between hepatic vascular pathology and protein homeostasis in clinical practice.

## 1 Introduction

Undernutrition is a deficient energy and nutrient status caused by insufficient dietary intake or malabsorption and can lead to adverse clinical outcomes [1]. Albumin and prealbumin are important markers for hepatic synthesis activity and body nutrient status. Hypoproteinemia is a condition characterized by abnormally low levels of proteins in the blood, particularly serum albumin. Albumin levels below 30 g/L are generally defined as hypoproteinemia, with normal reference values in healthy adults ranging from 35 to 50 g/L. Serum protein levels may drop below normal if the body is in a state of negative nitrogen balance for an extended period. Inadequate nitrogen intake can deplete the body’s protein stores, which include serum proteins like albumin and globulins, leading to hypoproteinemia [2].

The digestive process separates the macro and micronutrients from food into simple sugars, amino acids, fatty acids, vitamins, and other nutrients. These nutrients are then transported to the liver through portal veins. The liver is responsible for the biochemical transformation of these nutrients into substances with useful physiological properties [3].

Hepatic parenchymal cells produce the water-soluble, globular glycoproteins albumin and prealbumin. Prealbumin is so called because it migrates ahead of albumin during protein electrophoresis. Prealbumin is a measure of the health of the liver’s synthesis pathways, its reserve capacity, and its nutritional status. Normal hypoproteinemia is typically treated with albumin infusion, nutritional supplementation, or the elimination of the underlying cause of the condition [4].

Clinicians may also face cases of hypoproteinemia that are particularly difficult to treat. To improve albumin levels actively and symptomatically, clinicians need to first determine the pathological and physiological causes, which necessitates a series of examinations and tests and differentiation through multidisciplinary analyses.

We present a case of atypical-type undernutrition in a patient with progressive portal thrombosis and reduced blood inflow into the liver from portal veins, resulting in prolonged hypoproteinemia. This phenomenon has rarely been reported in the literature. In this article, we have focused on providing a theoretical basis for the diagnosis and treatment of atypical hypoproteinemia caused by progressive portal thrombosis and cavernous transformation of the portal vein (CTPV).

### 1.1 Case details

A 48-year-old male patient presented at our department of the hospital, with a chief complaint of persistent tachypnea lasting for more than one month. Notably, he had previously been diagnosed with cirrhosis 6 years prior, a condition initially identified during medical care subsequent to a motor vehicle accident in 2016. At that time, the patient exhibited no discernible symptoms of cirrhosis such as abdominal distension, asthenia, aversion to oily foods, anorexia, or any other related discomfort. Furthermore, there was an absence of subsequent medical evaluations or therapeutic interventions specifically targeting cirrhosis.

In the year 2020, the patient experienced a sudden aversion to oily foods without any apparent inciting factors; however, his overall dietary intake remained unaltered, and he did not manifest symptoms such as diarrhea or any associated discomfort. Remarkably, the patient did not seek further medical attention, nor did he pursue a comprehensive diagnostic workup or therapeutic intervention despite this notable change in his dietary preferences.

Over the passage of time, the patient exhibited a progressive state of emaciation, leading to a substantial reduction in body weight, amounting to approximately 15 kg from the year 2020 to the current date when he presented himself for medical evaluation at our facility. In April 2022, the patient reported an episode of tachypnea, which occurred spontaneously without any readily discernible precipitating factors. Remarkably, the patient denied experiencing symptoms such as aversion to cold, fever, recurrent fever, night sweats, cough, expectoration, nausea, vomiting, abdominal pain, abdominal distension, diarrhea, or any other associated discomfort during this period.

The patient had a previously unremarkable medical history, characterized by good health. In 2018, he underwent extracorporeal lithotripsy to address a “left kidney calculus.” Notably, the patient had a history of smoking spanning more than two decades, with a daily consumption of approximately 20 cigarettes. It is pertinent to mention that he successfully ceased smoking over nine months prior to the current medical evaluation. Additionally, the patient reported occasional, modest alcohol consumption, with no other noteworthy clinical features or medical history of significance.

The patient presented to our medical facility seeking further assessment and treatment. Results from auxiliary examinations revealed pertinent findings. Abdominal computed tomography (CT) imaging demonstrated a reduction in parenchymal density within the peripheral regions of both the left and right liver lobes, indicative of potential hepatic impairment or uneven perfusion. Notably, the liver exhibited substantial morphological changes in comparison to imaging conducted in 2016, characterized by diminished density, attenuated enhancement, and the presence of enlarged lesions.

Furthermore, the intrahepatic branches of both the portal vein and hepatic vein displayed a reduction in caliber, with certain branches exhibiting poor visibility. Additionally, there was an enlargement of the main portal vein by 1.5 cm, accompanied by the observation of numerous tortuous vascular shadows within the hepatic portal. These collective radiological findings strongly supported a diagnosis of CTPV, concurrently with the presence of cirrhosis and splenomegaly.

**Figure 1:**
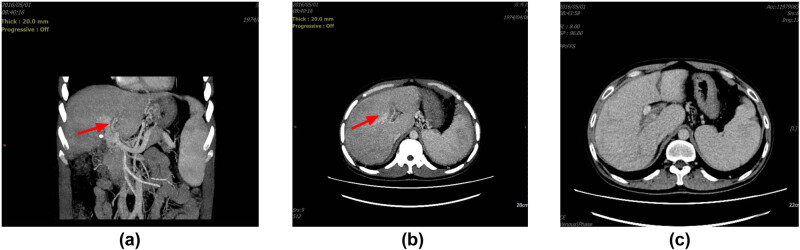
Imaging examination: a–c: In 2016, imaging revealed mild stenosis in the main portal vein. There was a conspicuous presence of collateral branches (indicated by the red arrow), facilitating the inflow of blood into the liver during the portal venous phase. Furthermore, the enhancement patterns observed in both the liver and spleen appeared uniform and essentially consistent.

**Figure 2:**
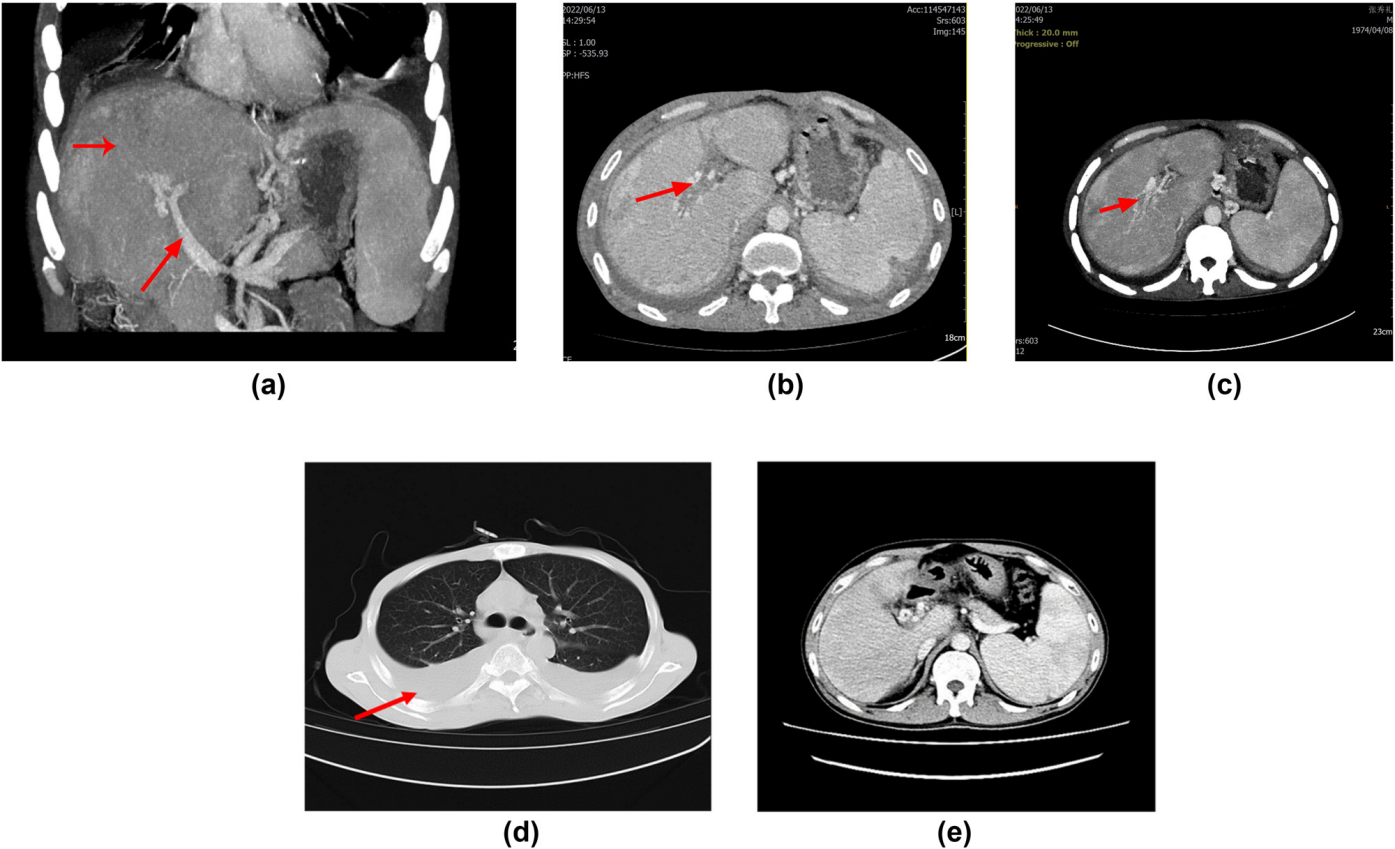
Imaging examination: (a) During reexamination in 2022: Significant worsening of main portal vein stenosis (indicated by the red arrow) with partial intrahepatic branches essentially having disappeared. In the portal venous phase of CT, significantly lower enhancement in most of the liver than in the spleen (indicated by the red arrow), significantly more aggravated portal hypertension, significantly thicker varices in the esophagus and stomach fundus, and a thicker splenorenal venous shunt. (b) The portal vein area shows thrombosis (indicated by the red arrow). (c) Spongy changes in the portal vein area (indicated by the red arrow). (d and e) Bilateral pleural effusion with no obvious signs of ascites.

The results of the thoracic CT examination revealed the presence of bilateral pleural effusion, with a depth of 6.0 cm in the right pleural space and 3.7 cm in the left pleural space (as illustrated in Figures 1 and 2). A gastroscopy procedure, conducted on June 7, 2022, unveiled the presence of moderate esophageal varices. It is noteworthy that these varices were classified as moderate in severity, with the absence of a red color sign, signifying an absence of active bleeding. Hepatic vascular ultrasound studies indicated the patency of the portal vein, hepatic vein, and inferior vena cava, suggesting normal vascular flow within the hepatic system. Cardiac color Doppler ultrasound findings revealed evidence of poorly coordinated ventricular wall motion in the heart, alongside mild regurgitation of both the mitral and tricuspid valves. Additionally, and weaker left ventricular diastolic function. The ejection fraction (EF) was 58%. Lastly, thyroid ultrasound investigations yielded unremarkable findings.

Laboratory investigation results were as follows: White blood cell (WBC): 9.7 × 10^9^/L (the maximum value, 12.79, was on June 7), neutral ratio 78.7%, hemoglobin (Hb) 100 g/L; procalcitonin 0.09 ng/mL, C-reactive protein (CRP) 72.87 mg/L; urine routine: occult blood 3+, urobilinogen 1+, urine protein±. Negative stool occult blood. Hepatic function: albumin 32.3 g/L (June 17 has the minimum value 29.9 g/L), prealbumin 62 mg/L, gamma-glutamyl transferase (GGT) 104.1, alkaline phosphatase (ALP) 519.4, with no remarkable abnormality in other respects in hepatic function; immunoglobulin G (IgG) 29.6 g/L, immunoglobulin A (IgA) 8.82 g/L, total cholesterol 2.16 mmol/L, normal triglyceride (TG). Blood coagulation: prothrombin time (PT) 15.8 s; international normalized ratio (INR) 1.41; D-dimer 0.67 mg/L; B-type natriuretic peptide (BNP) 10.78 pg/mL; renal function and tumor marker cancer antigen 125 (CA-125) 168.8 U/mL. Tests for myocardial markers, 24-h urine protein, anemia three items, thyroid function, hepatitis etiology, complete set of tests for autoimmune antibodies, and hepatitis antibody were all negative. K light chain 26.4 g/L, λ light chain 16.6 g/L. Purified protein derivative (PPD) test results were negative. Routine biochemistry analysis of pleural fluid showed exudate (Table 1).

**Table 1:** Laboratory investigation results

Investigation	Indicator	Results
Blood routine		
	WBC	9.7 × 10^9^/L (the maximum value, 12.79, was on June 7)
	Neutral ratio	78.7%
	Hb	100 g/L
	Procalcitonin	0.09 ng/mL
	CRP	72.87 mg/L
Urine routine		
	Occult blood	+++
	Urobilinogen	+
	Urine protein	±
Stool for routine		
	Stool occult blood	−
Hepatic function		
	Albumin	32.3 g/L (June 17 has the minimum value of 29.9 g/L)
	Prealbumin	62 mg/L
	GGT	104.1
	ALP	519.4
	Other respects in hepatic function	Negative
Immune globulin		
	IgG	29.6 g/L
	IgA	8.82 g/L
Blood fat		
	Total cholesterol	2.16 mmol/L
	TG	Negative
Blood coagulation		
	PT	15.8 s
	INR	1.41
	D-dimer	0.67 mg/L
Other investigation		
	BNP	10.78 pg/mL
	CA-125	168.8 U/mL
	K Light chain	26.4 g/L
	λ Light chain	16.6 g/L
	Tests for myocardial marker	26.4 g/L
	24-h urine protein	16.6 g/L
	Anemia three items	Negative
	Thyroid function	Negative
	Hepatitis etiology, complete set of tests for autoimmune antibodies, and hepatitis antibody	Negative
	PPD	Negative

**Figure 3:**
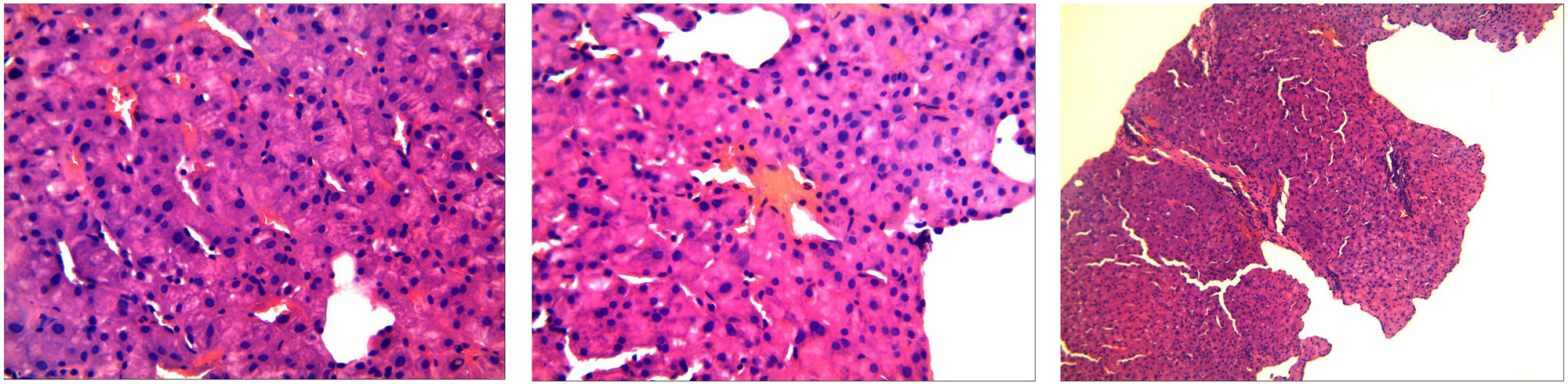
Hepatic pathology: Essentially normal liver tissue structure. Turbid and swollen hepatocytes. A small amount of pigment granule deposition in hepatocytes. Scattered punctate necrosis in the liver parenchyma. Narrowed sinusoids. Hepatic centrilobular vein not dilated. Normal shape and number of small bile ducts in the portal area. A small amount of chronic inflammatory cell infiltration in interstitial substance. No visible fibrous septa (hematoxylin and eosin [H&E] staining, ×100-fold).

Other tests: **Liver biopsy results**: Mild chronic liver tissue impairment. **Immunolabeling:** CD34 (transmembrane phosphoglycoprotein encoded by the CD34 gene in humans) (Focus +). CK19 (cytokeratin fragment 19) (bile ducts +). HBsAg (Hepatitis B surface antigen) (−). Normal liver tissue structure. Turbid and swollen hepatocytes. A small amount of pigment granule deposition in hepatocytes. Scattered punctate necrosis in liver parenchyma. Narrowed sinusoids. Not dilated hepatic centrilobular vein. Normal in shape and number of bile ducts in the portal area. A small amount of chronic inflammatory cell infiltration in the interstitial substance. No visible fibrous septa. No definite changes prompting cirrhosis (Figure 3). **Bone marrow (BM) slide test results:** Notable proliferation of BM nucleated cells with active BM hyperplasia and prominent erythrocyte proliferation indicated by pathology. **Genetic testing results:** Results of genetic testing by a third-party-accredited laboratory showed no abnormality in congenital-anemia-relevant hemolysis and paroxysmal nocturnal hemoglobinuria. (These tests were performed by SINO-US Diagnostics Lab, Tianjin, China on June 30, 2022).

## 2 Diagnosis and treatment for an integrated nutrition solution

Upon consultation, the patient, with a height of 165 cm and an initial weight of 45 kg, reported a substantial weight loss of 4 kg within a span of 2 weeks, representing a significant 8.16% reduction in body weight. Consequently, the body mass index (BMI) was calculated to be 16.5 kg/m². Clinical examination revealed a conspicuous diminishment of subcutaneous adipose tissue and muscle mass across various anatomical regions of the body. Notably, there was an absence of edema in both lower extremities. A comprehensive dietary assessment conducted by a nutritionist, encompassing a 24-hour observation of the patient’s customary daily food intake, estimated an energy intake of approximately 1,200 kcal and a protein intake of 52 g throughout the course of a typical day.

**Nutrition screening:** The patient received a score of 4 based on the Nutrition Risk Screening 2002 [5], indicating that he was at risk of malnutrition.

**Nutrition assessment:** Utilizing the criteria established by the Global Leadership Initiative on Malnutrition (GLIM) [6], the patient exhibited a low BMI of 16.5 kg/m², aligning with the phenotypical criteria. Furthermore, the evaluation of etiological criteria revealed a notable disease burden. Considering these factors in conjunction, the patient’s nutritional status was assessed as severe malnutrition, in accordance with the GLIM guidelines.

**Nutrition diagnosis:** Severe malnutrition, protein-energy undernutrition.

**Nutrition treatment:** Target energy was started at 1,500 kcal/day and gradually increased to 1,800 kcal/day if it was tolerated, with a target protein of 60–90 g. Apart from normal oral intake, supplementary nutritional preparations (300–500 kcal/day) were added based on the patient’s oral dietary intake.

**Nutrition status follow-up:** After nutritional interventions, prealbumin was checked every other day, and liver function and albumin were reviewed two weeks later, with close monitoring of the patient’s gastrointestinal symptoms.

Clinical diagnosis: 1. Severe malnutrition. 2. Non-cirrhotic portal hypertension. 3. Bilateral pleural effusion. 4. Hypoproteinemia. 5. Mild anemia. 6. With nutritional risk.


**Informed consent:** Informed consent has been obtained from all individuals included in this study.
**Ethical approval:** The research related to human use has been complied with all the relevant national regulations, institutional policies and in accordance with the tenets of the Helsinki Declaration, and has been approved by the authors’ institutional review board or equivalent committee.

## 3 Discussion

### 3.1 Cause analysis of undernutrition

Protein-energy malnutrition and micronutrient malnutrition are common causes of undernutrition. Micronutrient malnutrition manifests as a deficiency of micronutrients, such as vitamins and trace elements, which are required in minute quantities but are essential. Protein energy malnutrition manifests as insufficient energy and protein availability or absorption in the body [7]. In this instance, the patient’s food intake was normal, unchanged from his previous condition, and was devoid of food bias, anorexia, mismatched foods, or wasting disease. It was believed that he was suffering from malnutrition due to a lack of protein.

Causes of hypoproteinemia: Serum prealbumin, a glycoprotein produced in the liver, earns its name from its position in serum protein electrophoresis, where it precedes albumin. Notably, prealbumin boasts an exceptionally brief half-life of approximately 1.9 days. This distinctive characteristic endows prealbumin with the ability to provide a highly accurate reflection of recent overall nutritional status and the liver’s capacity for protein synthesis and secretion. Furthermore, prealbumin serves as a valuable tool for the early detection of liver function impairment, disease-related alterations, and prognosis assessment. Its utility extends to identifying early signs of malnutrition even in patients afflicted by non-malnutritional cirrhosis, making it a versatile marker in clinical contexts [8].

The liver can synthesize about 12–20 g of albumin per day with a half-life of about 21 days [2]. There are two types of albumin decrease: acute and chronic. The most common causes of acute albumin decrease are acute massive bleeding or severe burns that result in plasma loss. The causes of chronic albumin decrease are as follows:

1. Hepatic synthetic dysfunction. Hepatocyte degeneration and necrosis caused by various types of viral hepatitis or other hepatitis, liver cirrhosis, liver failure, liver cancer, and so on can interfere with hepatic function in albumin synthesis, followed by a rapid drop in albumin in the human body that can reach a significant total drop in about 10 days [9].
2. Undernutrition. The synthesis of albumin by the liver relies on the absorption of dietary protein and other nutrients. In instances of protein malnutrition or impaired nutrient absorption, hepatic parenchymal cells face a deficit of essential components necessary for albumin synthesis, ultimately resulting in a failure of albumin production. Consequently, serum albumin levels in the human body decline. Several scenarios exemplify this phenomenon: 1. Inadequate dietary intake: Reduced food consumption, as seen in fasting, consumption of low-nutrient foods, dysphagia, anorexia nervosa, selective eating habits, and dietary biases, can all lead to insufficient protein intake. This limitation in nutrient availability hampers the liver’s ability to synthesize albumin. 2. Gastrointestinal disorders: Various gastrointestinal conditions, such as small intestinal mucosal dysplasia, short bowel syndrome, fistulas in the small intestine or colon, gastric cancer, intestinal cancer, liver cancer, and portal hypertension, can impede the intake or absorption of essential proteins and nutrients. In such cases, the liver lacks the necessary raw materials required for albumin production, resulting in a decline in albumin levels within the body [10].
3. Loss of protein. (1) Nephrogenic hypoproteinemia. In the initial phases of conditions such as nephrotic syndrome and glomerulonephritis, protein loss predominantly involves smaller and finer proteins. However, as these conditions progress, proteinuria may encompass a wider range of protein types. In the later stages, it is possible for albumin levels to drop significantly, potentially falling below 10 g/L. When the concentration of albumin in the bloodstream falls below 20 g/L, it results in reduced colloid osmotic pressure. This decrease in colloid osmotic pressure has significant clinical implications, as it leads to the development of edema in the human body. (2) Low albumin levels can occur in patients with a variety of conditions, including severe burns, acute massive hemorrhage, and others where there is a significant loss of protein. (3) Large amounts of protein can also be lost due to hydrothorax and ascites. This can lead to hypoproteinemia, which in turn aggravates ascites. Later-stage renal impairment can further exacerbate protein loss. (4) Intestine-relevant protein loss: inflammatory intestinal disease, lymphangiectasia, autoimmune intestinal disease (for instance, Ménétrier disease which is associated with autoimmune pancreatitis) [11], protein-losing enteropathy [12], eosinophilic gastroenteritis, and so on.
4. Hypoproteinemia can also be caused by other chronic and consumptive diseases, such as tuberculosis and hyperthyroidism. Hypoproteinemia can develop as a result of excessive protein consumption or the negative effects on dietary intake caused by local malignant tumors in the lung, liver, kidney, etc., or systemic malignant tumors.
5. Pregnancy causes a drop in albumin levels, which is most noticeable in the third trimester. Albumin loss can also occur in extremely rare cases such as congenital albumin deficiency (where patients almost completely lack albumin in their blood but do not experience edema). Nutrition is less of a factor in hypoproteinemia than the underlying physiological stress caused by disease or trauma-related inflammation [13].

In addition to disease factors, malnutrition can also be caused by social and environmental factors. Poverty, living conditions, and other socio-economic factors have all been shown to affect the nutritional status of school-age children either directly or indirectly [14]. The most consistent factors associated with child malnutrition are maternal education, household income, maternal nutritional status, age of the child, availability of home health facilities, family size, family birth order, and birth weight of the child [15].

In summary, the concurrent reductions in albumin and prealbumin levels can serve as potential indicators of malnutrition and hepatic synthetic impairment. Through a comprehensive evaluation by a nutritionist, we deduced the possible causes behind the decline in albumin and prealbumin levels in this patient. Notably, malnutrition arising from inadequate dietary intake was ruled out, as the patient exhibited normal food consumption and did not present with symptoms such as dysphagia, dietary aversions, anorexia, or related issues. Malabsorption and protein loss related to intestinal diseases were also eliminated as potential causes for the decline in albumin and prealbumin levels. This exclusion was based on the patient’s favorable gastrointestinal function, which was characterized by the absence of symptoms such as abdominal pain, abdominal distension, diarrhea, and constipation. Furthermore, the patient’s hepatic function, as evidenced by normal bilirubin and transaminase levels, led us to exclude cirrhosis as a contributing factor, a determination further supported by the findings from a liver biopsy. Although the patient did display mildly abnormal blood coagulation parameters, we attributed this anomaly to portal vein thrombosis-induced consumption rather than hepatic synthetic dysfunction.

In 2016, the patient underwent a CT scan that revealed mild stenosis of the main portal vein, liver impairment or inadequate perfusion (the CT showed decreased hepatic blood inflow), a barely perceptible portal vein (likely indicative of thrombosis), and multiple tortuous vascular shadows surrounding the hepatic portal. Based on the CT done in 2016, CTPV was considered likely [16]. In the patient’s 2022 CT scan, a notable deterioration in the condition was observed, particularly in the main portal vein where there was more pronounced stenosis. Furthermore, several intrahepatic branches of the portal vein had virtually disappeared. During the portal venous phase of the CT scan, the majority of liver enhancements were markedly reduced compared to those of the spleen. Concomitantly, there was a significant exacerbation of portal hypertension, as evidenced by thicker varices in the esophagus and stomach fundus, as well as a more prominent splenorenal venous shunt when compared to previous assessments. Additionally, splenomegaly, or enlargement of the spleen, was evident in the imaging findings.

The patient was diagnosed with CTPV, which led to progressive portal thrombosis and progressive aggravation of portal hypertension [17], reducing blood flow and nutrient uptake into the liver, and ultimately leading to hypoproteinemia.

### 3.2 Diagnosis by exclusion

(1) Exclusion of cardiac organic diseases and heart failure: The patient presented with tachypnea but no other symptoms, such as edema in the lower extremities, asthenia, lung rales, jugular venous regurgitation, etc. The patient’s BNP was normal, and after performing a cardiac ultrasound scan to rule out organic cardiac diseases, we found that his EF was 58%. Consequently, we ruled out heart failure and other organic cardiac conditions.
(2) Exclusion of cirrhosis: There was no fibrosis, pseudolobules, or other pathological evidence of cirrhosis in the patient’s liver biopsy.
(3) Exclusion of malignant tumors of thorax and abdomen: There were no gastrointestinal symptoms present, such as nausea, vomiting, diarrhea, or constipation. His chest and abdomen CT scans came back clear, as did his tumor markers and his gastroscopy, and he was found to have no tumor in his stomach. As a result, there was a lack of supporting evidence for this disease.
(4) Exclusion of tuberculosis: No classic tuberculous symptoms, like persistent fever or night sweats, were present in this patient. The sputum slide test and chest CT both came back negative for tuberculosis.
(5) Exclusion of congenital hemolytic anemia and paroxysmal nocturnal hemoglobinuria: The patient had hypochromic microcytic anemia, a mild form of anemia. There was no evidence of fecal occult blood, ferritin, folic acid, and vitamin B12 levels for anemia, and bilirubin and hepatic function were both within normal ranges. Despite a positive urine occult blood and urobilinogen test, the patient’s urine bilirubin result was negative. Previous lithotripsy for renal calculi was linked to the patient’s urinary occult blood. In addition, genetic testing by an outside, reputable lab showed no indication of a problem with hemolysis or paroxysmal nocturnal hemoglobinuria, both of which are linked to congenital anemia. So, we eliminated paroxysmal nocturnal hemoglobinuria and congenital hemolytic anemia as possible causes.
(6) Exclusion of hyperthyroidism: The patient’s thyroid function and thyroid ultrasound both came back normal.

**Figure 4:**
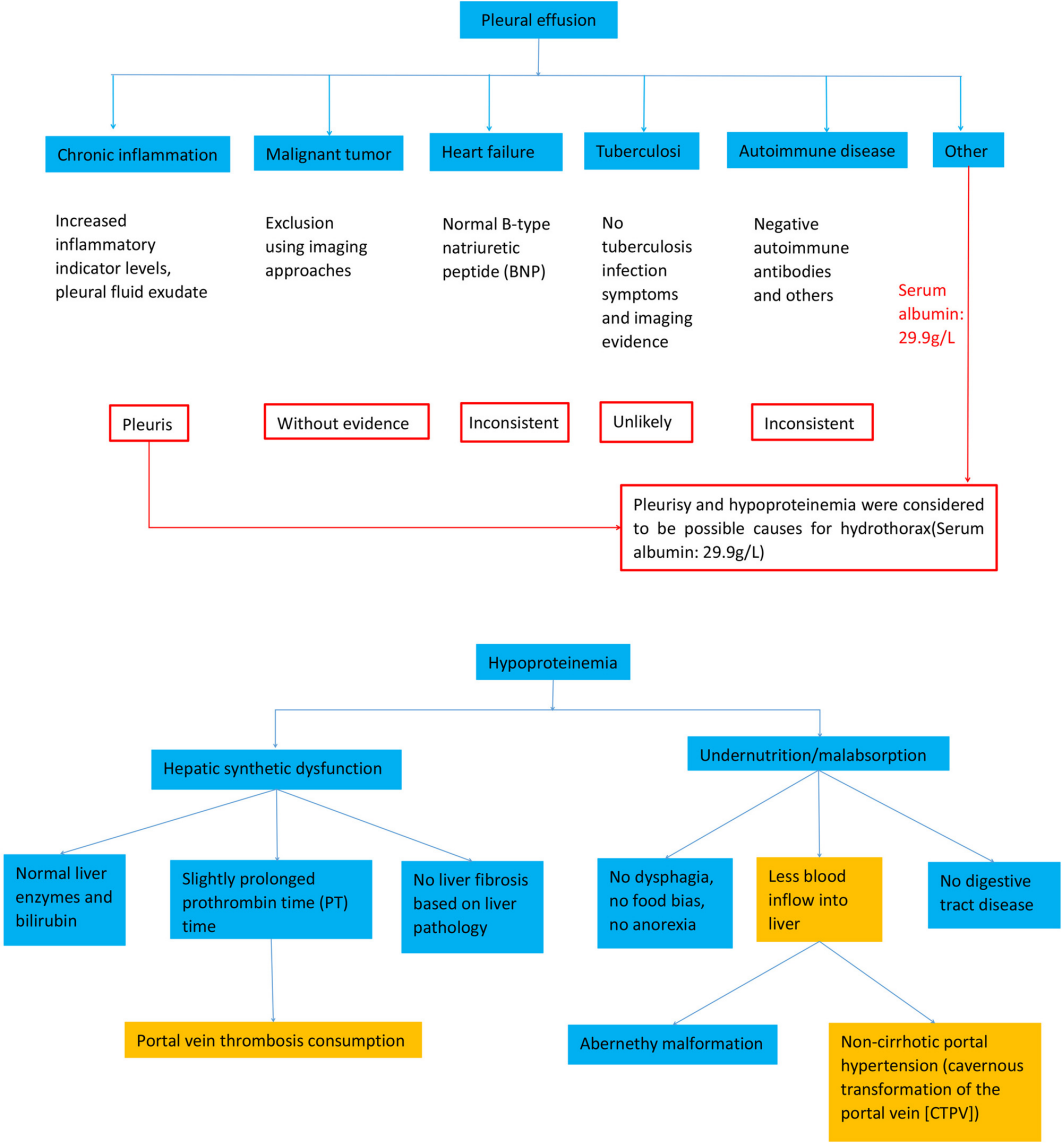
Mind map of the diagnostic procedure.

Figure 4 displays a mind map of the diagnostic procedure.

### 3.3 Treatment with concurrent verification

Following intravenous administration of 20% human albumin and compound amino acids (20AA) within our department, a notable improvement was observed in the patient’s condition. Specifically, there was a significant increase in serum albumin levels, rising from 29.9 g/L on June 17 to 33.9 g/L on June 21. This intervention also led to a reduction in hydrothorax, as well as relief from symptoms of cardiac exhaustion and tachypnea. However, it is worth noting that the patient’s symptoms worsened after discontinuing this intravenous infusion. This observation underscores the effectiveness of parenteral nutrition in rapidly increasing albumin levels and addressing the patient’s clinical presentation when compared to enteral nutrition, suggesting a more immediate and efficacious response to this mode of nutritional support.

### 3.4 Proposed treatment regimen for this patient

(1) Anticoagulation for early portal vein thrombosis. Unfortunately, the patient missed the optimal window for initiating anticoagulation therapy, as several years had elapsed since his initial diagnosis in 2016. Additionally, the patient was not a candidate for transjugular intrahepatic portosystemic shunt treatment. (2) Intravenous infusion of albumin and amino acids. It is important to note that albumin infusion does not exacerbate portal pressure. Early intravenous administration of albumin in appropriate quantities was shown to provide significant benefits, even in cases where cirrhosis developed subsequently [18]. (3) Adopt a dietary regimen characterized by smaller, more frequent meals, emphasizing a high-protein diet. (4) Wait for gradual compensation of hepatic artery. (5) Liver transplantation.

### 3.5 Chinese and international literature

Portal vein obstruction is the primary cause of CTPV-induced portal hypertension [17]. CTPV-induced portal hypertension can be divided into congenital and acquired categories. Malformations of the portal vein are the most common cause of portal hypertension due to congenital CTPV. Congenital atresia of the umbilical vein can also result from the involvement of the portal vein. Acquired CTPV-induced portal hypertension is caused by a local inflammatory state, such as portal phlebitis, pancreatitis, peritonitis, cholecystolithiasis, hepatic echinococcosis, and surgical trauma, or it is caused by thrombosis resulting from local tumor infiltration or other causes [19]. Some researchers have hypothesized that thrombophilia is the underlying cause of portal vein thrombosis [20]. In 2016, a CT scan showed signs of possible venous thrombosis in this patient. Over the course of 6 years, CTPV formed in the collateral circulation, blocking portal blood flow. There is currently a gap in the literature concerning the relationship between portal vein thrombosis and CTPV and hypoproteinemia.

The portal system transports nutrients from the digestive tract to the liver, where they are metabolized. This case report describes a patient who developed a progressive aggravated thrombosis in the main portal veins and their intrahepatic branches, causing a reduction in portal blood inflow into the liver, with the majority of this blood directly returning to the systemic circulation via the shunt. In this context, the compromised liver function led to an inability to acquire an adequate supply of essential substrates required for the synthesis of various proteins. Consequently, the nutritional components derived from food became inefficiently utilized, culminating in the development of hypoproteinemia, malnutrition, emaciation, and pleural effusion. It is noteworthy that the existing literature does not currently provide insights into the specific relationship between reduced blood inflow from the portal veins into the liver and the occurrence of hypoproteinemia. As a result, there is a paucity of documented knowledge on this particular aspect of the condition. We will continue to pay close attention to literature in this area, thereby facilitating the development of more effective strategies for addressing hypoproteinemia and malnutrition in patients with similar clinical profiles.

## 4 Conclusion

Vigilance for the development of hypoproteinemia holds paramount importance in the management of patients with progressive portal vein thrombosis complicated by CTPV. The prompt identification and implementation of effective interventions aimed at rectifying hypoproteinemia can yield substantial improvements in clinical outcomes. Moreover, the possibility of reduced hepatic blood flow should be meticulously considered as a potential underlying factor in instances of unexplained hypoproteinemia.
